# Functional dissection of HCMV gB’s autonomous fusion activity provides insights into how polymorphisms in clinical isolates confer distinct characteristics

**DOI:** 10.1128/mbio.00388-26

**Published:** 2026-07-13

**Authors:** Xiaohan Chen, Barbara Kropff, Jiawen Li, Heinrich Sticht, Madlen Mollik, Marie Sieger, Fulvia Ferrazzi, Irene Görzer, Michael Mach, Marco Thomas

**Affiliations:** 1 Harald zur Hausen Institute of Virology, Universitätsklinikum Erlangen, Friedrich-Alexander University Erlangen-Nürnberg, Erlangen, Germany; 2 Institute of Biochemistry, Friedrich-Alexander-Universität Erlangen-Nürnberg 9171, Erlangen, Germany; 3 Center for Virology, Medical University of Vienna 27271, Vienna, Austria; 4 Department of Nephropathology, Institute of Pathology, Universitätsklinikum Erlangen, Friedrich-Alexander-Universität Erlangen-Nürnberg, Erlangen, Germany; 5 Institute of Pathology, Universitätsklinikum Erlangen, Friedrich-Alexander-Universität Erlangen-Nürnberg, Erlangen, Germany; Duke University School of Medicine, Durham, North Carolina, USA

**Keywords:** human cytomegalovirus, glycoprotein B, gB, gB-genotypes, gB polymorphisms, gH/gL-independent fusion, cell-cell fusion, mechanism of pre- to post-fusion transition

## Abstract

Glycoprotein B (gB) serves as the viral fusogen facilitating entry and cell-to-cell spread of human cytomegalovirus (HCMV). Fusion is initiated when gH/gL-containing complexes bind to their receptors, leading to gB’s transition from a metastable prefusion to a stable postfusion conformation. We recently demonstrated that substituting or truncating the carboxy-terminal domain of gB renders it intrinsically fusion-competent; however, the precise mechanisms of gB ectodomain reorganization—likely shared by both gH/gL-dependent and -independent fusion processes—remain unclear. In this study, a dual split protein (DSP)-based cell-cell fusion assay was used to assess the gH/gL-independent fusion activities across five major HCMV gB-genotypes (gB1–gB5). Our results show that gB of VR1814 (gB3) exhibits superior autonomous fusion activity compared to TB40/E (gB1) and AD169 (gB2), while gB from C194A (gB4) and UKNEQAS1 (gB5) were fusion-incompetent. Through domain-swapping and site-directed mutagenesis, we identified substitutions that enhance or abrogate fusion and mapped them onto the available pre- and post-fusion structures of gB. Our analysis uncovered a complex network of intra- and inter-domain interactions involving structural domains I, IV, and V of gB, which are crucial for fusion regulation. Functional characterization of gB polymorphisms from clinical HCMV isolates demonstrated opposing impacts, with some variants remaining inactive, while others conferred fusion competence. Collectively, our findings mechanistically elucidate how specific substitutions within the gB ectodomain of particular HCMV strains markedly alter gH/gL-independent fusogenicity. These insights have profound implications for developing diagnostic tools to detect functionally relevant gB polymorphisms and for optimizing vaccine design.

**IMPORTANCE:**

Human cytomegalovirus (HCMV) establishes lifelong, predominantly asymptomatic infections but remains a major cause of morbidity and mortality in immunocompromised individuals and newborns. This study elucidates the mechanisms driving the structural reorganization of the gB ectodomain, which likely regulate both gH/gL-independent and -dependent fusion activities across gB-genotypes gB1–gB5. Through a combinatorial *in silico* and functional approach, we determined that C-terminally truncated gB is fusion-incompetent when expressed alone. However, clinical isolates evolved diverse fusion-enhancing substitutions, which either destabilize the prefusion conformation or inhibit the formation of the postfusion conformation of gB, thus rendering the protein fusion-competent. The presence of these substitutions in isolates from diverse body compartments suggests a possible implication for HCMV pathogenicity and transmission. Assessing the gH/gL-independent cell-to-cell fusion activity of gB provides a valuable method for identifying functionally relevant polymorphisms, laying the foundation for further viral infection studies and guiding vaccine development.

## INTRODUCTION

Human cytomegalovirus (HCMV) is a highly prevalent betaherpesvirus and a major global health concern. Although infection is typically asymptomatic in immunocompetent adults, it poses substantial risks to immunocompromised individuals, including stem cell or solid organ transplant recipients and patients with acquired immune deficiency syndrome (AIDS) (1–3). As the leading cause of congenital viral infection, HCMV affects approximately 1 in 150 live-born infants worldwide (4). Approximately 10% of congenitally infected newborns are symptomatic at birth, and a subset of initially asymptomatic infants later develop long-term sequelae (5). HCMV is the primary infectious contributor to sensorineural hearing loss and is associated with a range of neurodevelopmental disorders in children (6). Current antiviral therapies are limited by toxicity and the emergence of drug-resistant strains (7), underscoring the need for effective prophylactic vaccination, which has so far achieved only limited success (8).

**Fig 1:**
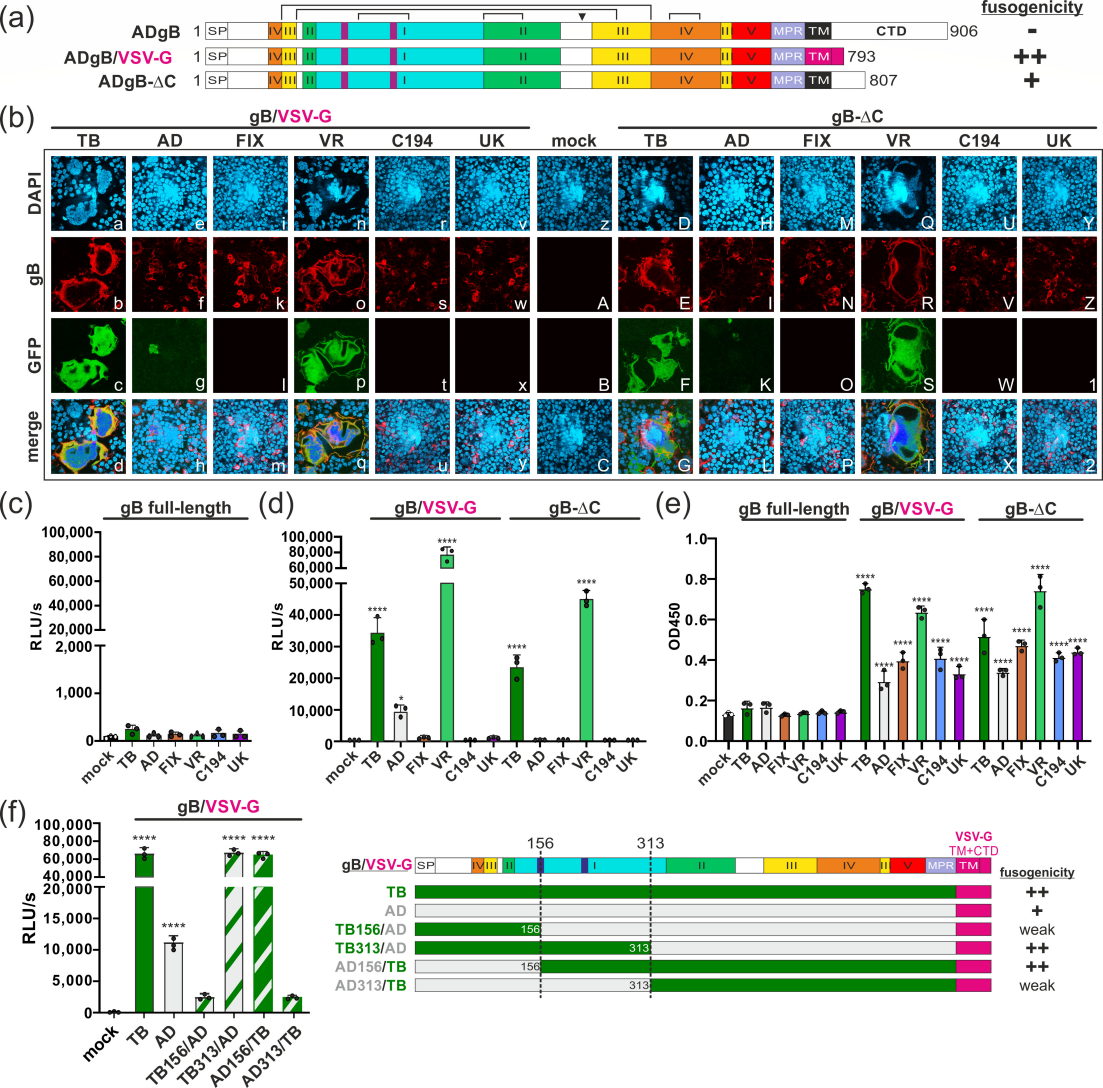
Glycoprotein B genotypes differ in their gH/gL-independent cell-cell fusion activity. (**a**) Linear representation of full-length glycoprotein B encoded by HCMV strain AD169 (ADgB) along with the fusion-competent constructs ADgB/VSV-G and ADgB-ΔC. Structural domains I–V are displayed in different colors in analogy to the crystal structure (19). Brackets in the top indicate disulfide bonds, and the arrowhead highlights the cleavage site of the cellular endopeptidase furin. SP, signal peptide; MPR, membrane-proximal region; TM, transmembrane domain; CTD, carboxy-terminal domain. (**b–f**) 293T-DSP-mix cells were transfected with vectors encoding either of the indicated gB constructs or an empty vector as a control (mock). ADgB/VSV-G and TBgB/VSV-G and chimeric proteins thereof were generated by using shared single-cutter enzymes; the breakpoints of the chimera are indicated by “156” and “313” in the schematic summarizing the results of the DSP assay (**f**). (**b**) Indirect immunofluorescence analysis. Following fixation and permeabilization, cell nuclei were stained with DAPI (first row). Expression and subcellular localization of gB were examined using confocal laser scanning microscopy upon staining with the anti-gB antibody 27–287 (second row, gB). Cell-cell fusion was assessed based on the GFP signal from the reconstituted DSP-reporter protein (third row, GFP), and the formation of multinucleated syncytia (fourth row, merge). (**c, d, and f**) Quantification of cell-cell fusion by bioluminescence following addition of the substrate EnduRen at 50 h (**c**) or 24 h (**d and f**) post-transfection. (**e**) Cell surface exposure of gB-constructs as evaluated by cELISA at 24 h post-transfection as described in the methods section. (**c–f**) Experiments were performed three times (three dots); columns depict the mean of biological triplicates ± standard deviations. Statistical analysis was performed by using one-way ANOVA using Bonferroni’s multiple comparison test, *: *P* < 0.1, ****: *P* < 0.0001. *P*-values refer to cells transfected with mock and were not statistically significant unless indicated.

Promising vaccine candidates include the virus-encoded glycoproteins required for the entry and cell-cell spread of the virus. While a trimeric complex composed of gH/gL/gO is required for entry into all cell types, the pentamer composed of gH/gL/UL128-131 mediates entry into epithelial, endothelial, and myeloid cells (9–11). In either case, it is believed that upon receptor binding, the signal is transferred to gB (gpUL55), a conserved class III fusion protein shared across the Herpesviridae (12–14). One plausible mechanism of fusion activation was recently provided by the Heldwein group, showing that the HCMV gB C-terminal Domain (CTD) restricts fusion until triggered by the gH cytoplasmic tail upon binding of the trimer to PDGFRα (15). In line with the critical regulatory role of the CTD, we demonstrated in our previous work that either replacing the transmembrane (TM) and CTD of gB with that of the structurally related vesicular stomatitis virus G protein (final construct named gB/VSV-G) or the truncation of the gB-CTD (hereafter named gB-ΔC) conferred intrinsic fusion capacity to HCMV gB (16, 17) (Fig. 1a). Whether during viral entry or cell-to-cell fusion, gB facilitates membrane fusion through major conformational transitions from a metastable prefusion state, involving unfolding, insertion of its hydrophobic fusion loops into the host cell membrane, retrimerization, and refolding of the gB protein into a low-energy postfusion form, ultimately resulting in membrane merger (14, 18, 19). However, the detailed mechanisms of these steps require experimental validation.

One major obstacle for vaccine development is the extensive variability documented for the various HCMV envelope glycoproteins. Despite its relatively good conservation, gB is known to cluster into seven genotypes (gB1 through gB7), defined by sequence polymorphisms at the gB N-terminus and its internal cleavage site for the cellular endoprotease furin (20–22). The various gB-genotypes have been identified across multiple geographical regions, from various body fluids and different patient populations (23, 24). Moreover, mixed infections with multiple HCMV strains and intrahost-recombinations may occur within a single host (25, 26). This is why clear associations between any particular gB-genotype and disease severity have not yet been firmly established, and it remains unclear whether these genotypes differ in their respective fusion capacities (23, 24; and references therein).

Structural and biochemical studies have shown that the gB ectodomain is comprised of five domains (Dom I–V) that coordinate the dynamic rearrangements required for fusion (18, 19). Importantly, recent work has demonstrated that single amino acid changes can profoundly modulate gB-mediated fusion. The Dom I polymorphism D275Y of gB2 laboratory-adapted strain AD169 was identified as the cause of a hyper-fusogenic phenotype, marked by rapid virion entry, extensive syncytia formation, and strong activation of DNA damage signaling pathways (27). Another single amino acid substitution was identified in Dom IV at position 585 of gB3, where the glycine of the clinical isolate VR1814 was replaced by serine in the bacterial artificial chromosome derived from this isolate, named FIX-BAC. This G585S polymorphism was shown to have profound effects on viral entry and cell-cell fusion capability (28). Understanding the molecular mechanisms underlying these strain-specific polymorphisms is crucial for elucidating the determinants of viral entry, tissue tropism, pathogenicity, and immune control, as well as for guiding the development of vaccines and antiviral strategies.

In this study, we conducted a structure-function analysis of the autonomous fusion activity of HCMV gB encoded by selected representatives from the five major HCMV gB-genotypes (gB1–gB5). In addition to gB encoded by “AD169” (gB2), isolated from adenoids (29), and “VR1814” (gB3), isolated from the cervical secretions of a pregnant woman (30), we included representatives of gB-genotypes 1, 4, and 5. We chose strain “TB40/E” (gB1), isolated from the throat wash of a bone marrow transplant recipient, “C194A” (gB4) from a solid organ transplant recipient, and “UKNEQAS1” (gB5) from the urine of a congenitally infected infant, respectively (21, 31). Through domain swapping and targeted site-directed mutagenesis, we aimed to identify key residues that either enhance or abolish fusion in the absence of gH/gL, utilizing the well-established dual split protein (DSP) cell-cell fusion assay (16, 32). Through a detailed structure-function dissection, we explain mechanistically how key polymorphisms within the gB ectodomain influence its autonomous fusion competence, thereby informing future vaccine and antiviral strategies to interfere with this critical step of infection.

## RESULTS

### gB-genotypes differ in their gH/gL-independent cell-cell fusion activity

To compare the intrinsic fusion capacity, gB encoded by “TB40/E” (TB, gB1), “AD169” (AD, gB2), “FIX-BAC” and “VR1814” (FIX and VR, gB3), “C194A” (C194, gB4), and “UKNEQAS1” (UK, gB5) was cloned either as a full-length protein or as their modified derivatives gB/VSV-G or gB-ΔC. Next, synthesis of the newly generated constructs was assessed using confocal microscopy from transfected 293T-DSP-mix cells, which confirmed comparable protein expression and subcellular localization of the gB/VSV-G or gB-ΔC constructs (Fig. 1b, panels in the second row). In accordance with our previous publications, TB induced robust fusion activity, as demonstrated by a significantly higher number of GFP-positive cells and extensive multinucleated syncytium formation (Fig. 1b, panels c and F) (16, 17). In contrast, AD produced fewer GFP-positive cells and smaller syncytia (Fig. 1b, panels g and K). Consistent with the findings of Tang et al., the substitution of FIX’s S585G converted the fusion-deficient protein into the highly potent fusogen VR, which induced syncytia even larger than those formed by TB (Fig. 1b, compare panels l and p or O and S) (27). In contrast, C194 and UK did not induce notable cell-cell fusions in the absence of other viral proteins (Fig. 1b, panels t and W or x and 1).

For unbiased quantification of the cell-cell fusions, complementation of Renilla luciferase (RLuc) was assessed next. Consistent with previous findings (16, 17, 33), full-length gB of all genotypes was fusion-inactive (Fig. 1c). In contrast, fusion was detectable in the gB/VSV-G chimera and less pronounced upon truncation of the gB-CTD (Fig. 1d), which is in line with our previous observations with low-fusogenic ADgB-ΔC (16). In accordance with the immunofluorescence results, VR exhibited the strongest fusion activity, which was approximately 2-fold higher than TB and nearly 8-fold higher than AD. In contrast, FIX, C194, and UK did not yield significant RLuc activity in both the gB/VSV-G and the gB-ΔC context (Fig. 1d).

To assess whether variability in fusion activity was attributable to differences in cell surface display of the gB constructs, a cell-based ELISA (cELISA) was performed. Consistent with previous findings, full-length gB was scarcely detectable at the surface, whereas gB/VSV-G and gB-ΔC variants showed robust cell surface exposure (Fig. 1e) (16). Although surface expression differed among constructs, these differences did not correlate with fusion activity, as reported previously for ADgB/VSV-G and ADgB-ΔC (16). Based on these results, subsequent experiments were conducted under genotype-specific conditions to achieve optimal protein expression for each construct.

Full-length TBgB and the less fusogenic ADgB share a high pairwise sequence identity of 95.6% and a similarity of 97.4%. To elucidate the molecular mechanisms underlying their differences in fusion competency, a panel of domain-swap chimeras was generated. By employing shared single-cutter restriction enzymes for cloning, the N-terminal region of gB, either up to residue 156 or extending to residue 313, was swapped between the two gB variants within the context of the gB/VSV-G chimera. Strikingly, TB313/AD and AD156/TB retained robust fusion activity, comparable to that of parental TBgB/VSV-G, whereas TB156/AD and AD313/TB displayed markedly reduced fusion activity, which was even lower than that of ADgB/VSV-G (Fig. 1f). These findings suggest that polymorphisms within the N-terminus—comprising the signal peptide (SP; residues 1–22), the N-terminal domain (NTD; residues 23–88), and portions of Dom IV and III (residues 89–111)—and/or within Dom I (residues 133–343) may serve as critical determinants for the regulation of gB-mediated membrane fusion in the absence of gH/gL.

### The N-terminus alone is not responsible for the fusion deficiency of gB4 and gB5, but it contains fusion-regulating residues

**Fig 2:**
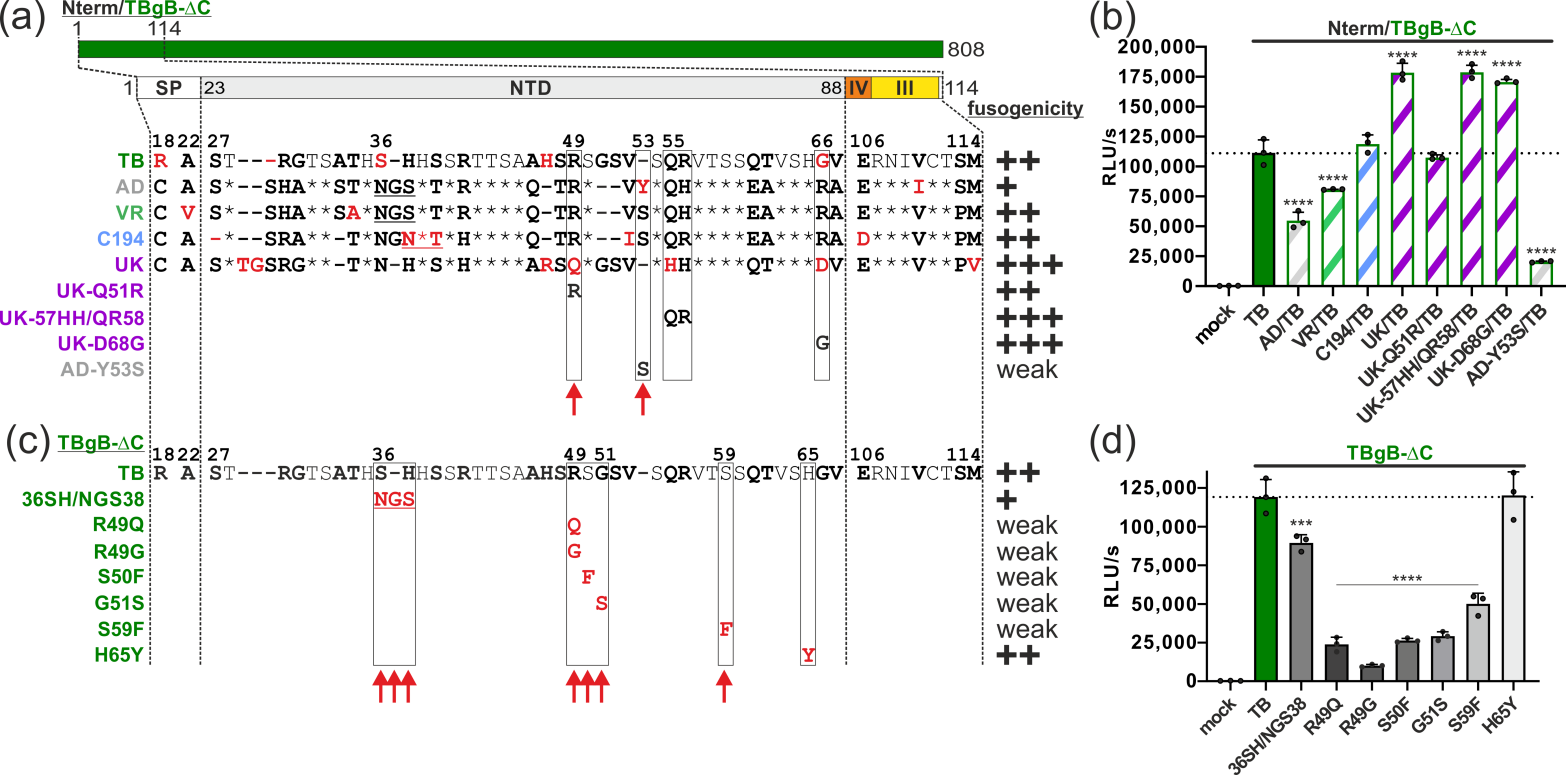
The highly heterogeneous N-terminus of gB regulates its fusion activity. (**a + c**) Schematic representation of the chimeric Nterm/TBgB-ΔC construct, showing the signal peptide (SP), N-terminal domain (NTD), and structural Domains III and IV up to residue 114, along with the respective amino acid sequences and positions of TBgB; note the offset in numbering for UK and AD. (**a**) The N-terminus (Nterm) encoded by residues 1–114 of TBgB-ΔC (TB, gB1) was replaced with the corresponding sequences from AD169 (AD, gB2), VR1814 (VR, gB3), C194A (C194, gB4), and UKNEQAS1 (UK, gB5), respectively, and point mutants thereof. Conserved residues are marked with asterisks, while divergent residues are shown in bold and predicted glycosylation motifs are underlined. Red highlighted residues are unique to individual genotypes (**a**) or polymorphisms identified in clinical isolates (**c**). (**a and c**) Analyzed mutations are boxed, and red arrows indicate residues identified as critical for fusion regulation. The “fusogenicity” column summarizes relative fusion activity determined by DSP assays, with the number of “+” symbols indicating the extent of fusion activity. (**b and d**) DSP luciferase reporter assays for the quantification of cell-cell fusion by bioluminescence are given in relative light units per second (RLU/s). Experiments were performed three times (three dots); columns depict the mean of biological triplicates ± standard deviations. The dotted line indicates the fusion activity of parental TBgB-ΔC. Statistical analysis was performed by using one-way ANOVA using Bonferroni’s multiple comparison test, ***: *P* < 0.001, ****: *P* < 0.0001. *P*-values refer to cells transfected with TBgB-ΔC and were not statistically significant unless indicated.

To assess the impact of the N-terminus (Nterm), a panel of chimeras was generated in which the SP, NTD, and Dom IV and III up to residue 114 of TBgB, were replaced with corresponding regions from gB2 to gB5 (Fig. 2a). All Nterm-swap constructs were fusion active as measured by the DSP assay (Fig. 2b). Since C194-Nterm worked properly in the context of TB, one can exclude that the lack of S27, the unique predicted glycosylation site 37NHT39 or the substitutions I50 or D104 as causes of C194gB’s fusion deficiency (red letters in Fig. 2a). The AD- and VR-Nterm/TB-chimera were 2-fold and 1.4-fold less active, respectively, while the UK-Nterm/TB-chimera demonstrated an up to 2-fold increase in activity compared to parental TBgB-ΔC. This suggests that unique residues of the N-termini of AD, VR, and UK are involved in regulating the gH/gL-independent fusion of gB.

To pinpoint the responsible N-terminal residues, three UK-specific sites Q51, H57/H58, and D68 were individually reverted to the corresponding TB residues (R49, Q55/R56, and G66). Only the Q51R exchange in UK reduced its fusion activity to the level of TBgB-ΔC, whereas 57HH/QR58 and D68G had no significant effect, identifying Q51 as a principal trigger for the hyperfusogenic activity of the UK-Nterm/TB swap construct. In contrast, the unique sites T29/G30, R49, and V116 in UK were negligible for fusion regulation (red letters in Fig. 2a). The AD-Nterm/TB swap construct, which was ~50% less active than the parental TBgB-ΔC, encodes the adjacent residue Y53, which can be found in approximately 43% of the gB2 strains. In contrast, this residue is absent in gB1 and gB5 strains, which were the most fusogenic constructs in this assay. In fact, the fusion activity of AD-Nterm/TB was further abrogated when Y53 was exchanged for serine as encoded by 57% of gB2 strains and all gB3 and gB4 strains, suggesting that Y53 partly restrains fusion in this context (Fig. 2b).

To further support the hypothesis that the N-terminus serves as a hot spot for fusion-affecting polymorphisms, we analyzed the impact of various substitutions identified in clinical isolates on gB’s fusion activity. Residue Q51 is fully conserved in all known gB5 strains and corresponds to Q49 in the gB1 strain PAV32 (AQN69508), which was isolated from the plasma of a transplant recipient. In contrast, over 97% of the published gB sequences feature an arginine at this position, and a glycine has been reported in “2vaginal8-11-2000” (AZB53158), “DUR12” (CAG7582488), and “PRO-66-mid82-ul55” (WMN01602), the latter isolated from a congenitally HCMV-infected patient (34). The adjacent S50 is conserved in 99% of all gB strains. Exceptions include “NL/Rot4/Nasal/2012” (AMJ53091.1) and “BE/48/2011” (AKI20319.1), both isolated from nasopharyngeal aspirates and possessing an F50 at this position. We further examined the positions corresponding to G51 in TB, which is substituted with S51 in the isolate “HANSCTR12” (AQN73719), obtained from the blood of a stem cell transplant recipient, and H65, which is replaced by Y65 in “BE/22/2010”(AKI08783), isolated from urine. DSP assays revealed that while the H65Y substitution had no effect, inserting the potential glycosylation site 36NGS38 from AD or VR significantly reduced fusion (Fig. 2d). Thus, glycosylation at 36NGS38 likely contributes to the reduced fusion activity observed in the AD- and VR-Nterm/TB chimeras (Fig. 2b). Similarly, all other polymorphisms from clinical isolates significantly impaired fusion activity. These findings collectively suggest that although NTD polymorphisms at positions corresponding to TBgB S36 through S59 can influence fusion efficiency, the NTD alone does not account for the fusion-inactive phenotypes of C194 and UK. This suggests that regions beyond the heterogeneous N-terminus primarily determine variation in gH/gL-independent fusogenicity across gB-genotypes.

### Dom I contains critical determinants of fusion regulation

**Fig 3:**
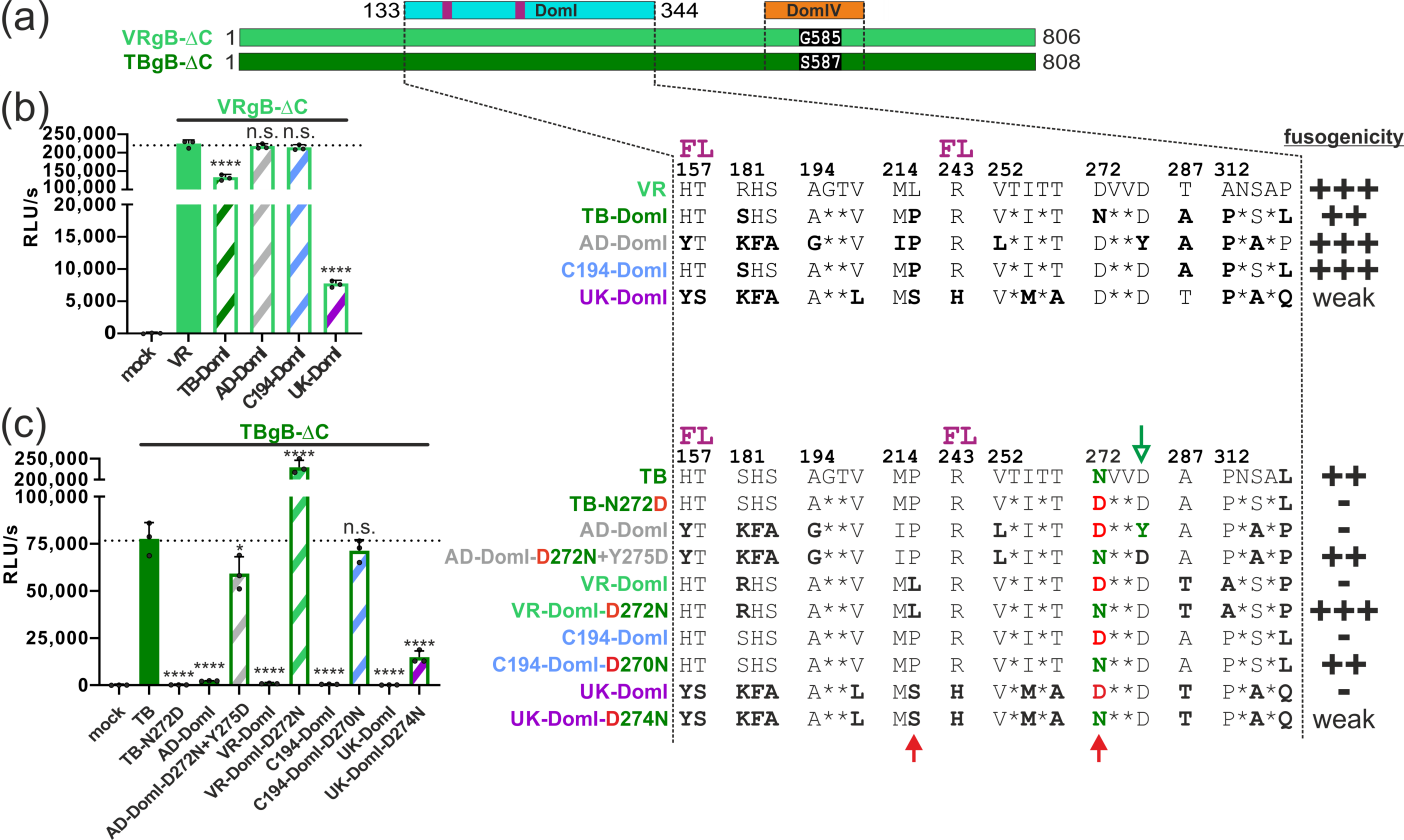
Domain I modulates the membrane fusion activity of various gB-genotypes. (**a**) Schematic representation of gB domain swap constructs. Residues 133–344 encoding structural Domain I (Dom I), including the fusion loops (FL) of VRgB-ΔC or TBgB-ΔC, were replaced with the corresponding region of other genotypes. (**b and c**) Left: 293T-DSP-mix cells were transfected with empty vector (mock) or plasmids encoding the indicated chimera or point mutants thereof. Cell-cell fusion activity was quantified by luciferase reporter assays and is given in RLUs per second. Experiments were performed three times (three dots); columns depict the mean of biological triplicates ± standard deviations. The dotted line indicates the fusion activity of parental VRgB-ΔC (**b**) or TBgB-ΔC (**c**), respectively. Statistical analysis was performed by using one-way ANOVA using Bonferroni´s multiple comparison test, *: *P* < 0.1, ****: *P* < 0.0001, n.s.: non-significant. *P*-values refer to cells transfected with VRgB-ΔC (**b**) or TBgB-ΔC (**c**), respectively. Right: sequence alignments highlight variable residues within Dom I. Conserved residues are marked with asterisks, and variable amino acids are highlighted in bold, with fusion-attenuating mutations indicated in red and fusion-enhancing mutations in green. Green arrow indicates the previously identified Y275 polymorphism of ADgB, while red arrows indicate the newly identified variations associated with altered fusogenicity; note the offset in numbering for C194 and UK. For the L/P/S polymorphism corresponding to TBgB’s position 215, see Fig. 5.

As the initial experiment revealed a critical involvement of residues 156–313, we next directed our attention to Dom I (residues 133–343), known for eliciting neutralizing antibodies (35). A panel of chimeric constructs was generated, in which Dom I from each gB-genotype was introduced into the backbones of the highly fusogenic VRgB-ΔC or the robustly fusogenic TBgB-ΔC (Fig. 3a). Replacing Dom I of VR with that from AD or C194 resulted in high fusion activity, whereas substitution with TB- or UK-Dom I led to a loss of fusion activity by approximately 1.7-fold or 30-fold, respectively (Fig. 3b). These results excluded the possibility that gB4-specific polymorphisms in Dom I cause the fusion defects of C194gB-constructs, while TB- or UK-specific Dom I substitutions contribute to fusion impairment.

Sequence alignment of all annotated gB sequences revealed that TBgB carries the unique D272N polymorphism in close proximity to the recently identified Y275 of ADgB, which is associated with a syncytium-forming (hyperfusogenic) phenotype (27). In fact, all DomI/TBgB-ΔC chimeras remained fusion-negative, except when TB’s unique N272 was introduced. This substitution restored the fusion activity of AD- and C194-Dom I to wild-type levels and increased VR-Dom I activity 3-fold compared to TBgB-ΔC (Fig. 3c). In line with the observation described above, UK’s fusion activity could only marginally be increased by N274, indicating that it contains additional negative regulators that account for the loss of its fusogenicity (Fig. 3c). These data demonstrate that in the absence of gH/gL, Dom I has genotype-specific roles in regulating gB’s fusogenicity and that TBgB-ΔC only fuses if N272 is present.

### N272 and G585 confer hyperfusogenicity to other gB-genotypes

**Fig 4:**
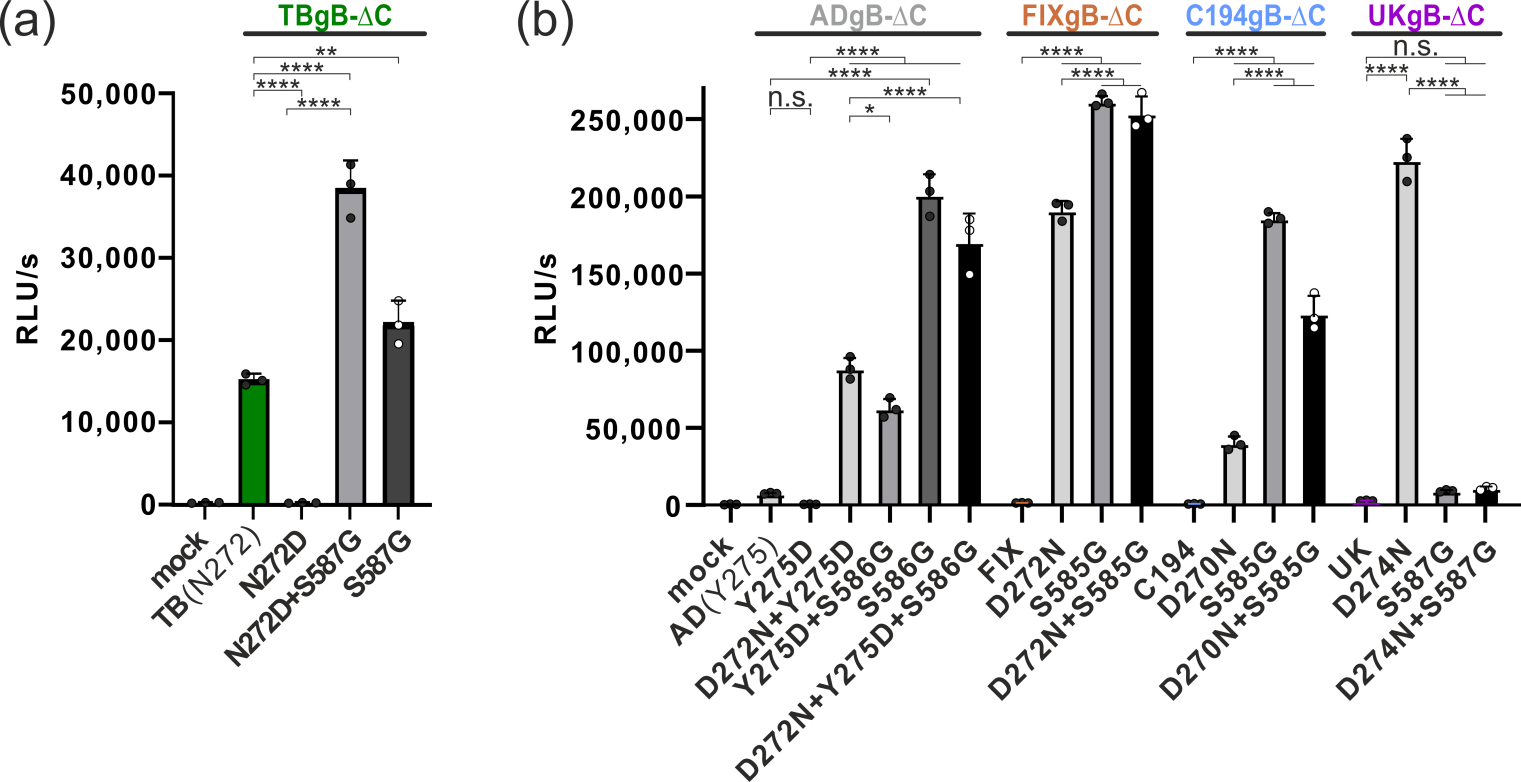
N272 and G585 confer hyperfusogenicity to all tested gB-genotypes. (**a and b**) Cell-cell fusion activities of gB-ΔC and its indicated derivatives. 293T-DSP-mix cells were transfected with an empty vector (mock) or plasmids expressing gB-ΔC variants encoding substitutions D272N or S585G alone or in combination (D272N + S585G). Note that the respective amino acid position varies slightly in the different gB-genotypes. Fusion activity was quantified by luciferase reporter assay and expressed as RLU/s. Experiments were performed three times (three dots); columns depict the mean of biological triplicates ± standard deviations. Statistical analysis was performed by using one-way ANOVA using Bonferroni’s multiple comparison test, *: *P* < 0.1, **: *P* < 0.01, ****: *P* < 0.0001, n.s.: non-significant.

Consistent with previous findings, our results indicate that the prototypical gB-ΔC—encoding the consensus residues D272, D275, and S585—is fusion-defective, as exemplified by TB-N272D, AD-Y275D, FIX, C194, and UK (Fig. 4a and b). However, when tested in the context of gB-ΔC, some strains carry syncytium-enhancing substitutions–N272 in TBgB, Y275 in ADgB, and G585 in VRgB—which, in part, correlate with enhanced *in vitro* syncytium formation when expressed in their authentic viral backbones, as shown for the AD169 and VR1814 strains (27, 28). We next compared their fusion activities to determine which of them is superior and if they remain functional across different gB-genotypes. In line with previous reports, cell-cell fusion assays confirmed that the typical gB2 construct AD-Y275D is fusion-defective but becomes fusogenic upon substitution with D275Y, as demonstrated by ADgB (Fig. 4b). In the same way, N272 or G585 consistently conferred hyperfusogenic phenotypes to all tested gB-genotypes, suggesting a conserved role in regulating the fusion activity of HCMV gB in the absence of gH/gL (Fig. 4b).

Comparing G585 and N272 reveals that G585 induces significantly higher fusion activity than N272 in TB, FIX, and C194, indicating that G585 plays a more significant role in enhancing fusion in these gB-genotypes (Fig. 4a and b). In contrast, in gB2, N272 was slightly more effective than G586, and both surpassed the activity of Y275 found in AD. Notably, the combination of G585 and N272 did not result in further increased fusion activity in TB, FIX, and C194 compared to the individual substitutions. Of note, gB2 was the only genotype where combining G586 with either Y275 or N272 led to an approximately 2-fold increase in fusion activity. Interestingly, UK was the only strain where D274N enhanced fusion more robustly than S587G, but again, combining both mutations did not result in further enhancement (Fig. 4b). These observations suggest that residues N272 and G585 independently function as positive regulators of gB-mediated autonomous membrane fusion across diverse gB-genotypes.

### gB5 evolved fusion-attenuating residue S217 and Dom IV loop 566TIKDST571

**Fig 5:**
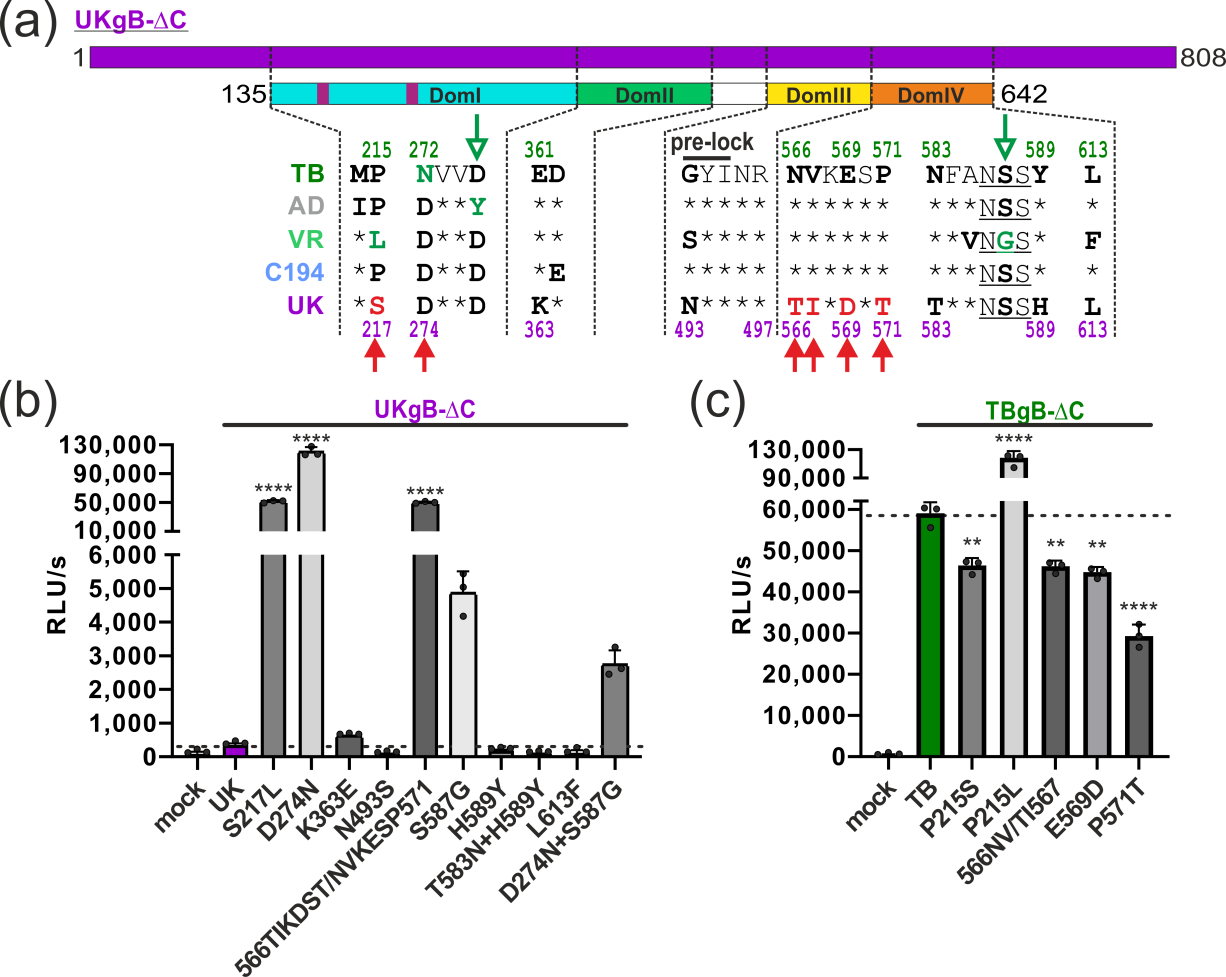
gB-genotype 5 encodes the characteristic fusion-attenuating residues S217 and 566TIKDST571. (**a**) Schematic representation of UKgB-ΔC highlighting Domains I–IV (residues 135–642). Sequence alignment of the corresponding regions from representative HCMV strains (TB, AD, VR, C194, and UK) shows conserved residues indicated with an asterisk. Variable amino acids are highlighted in bold, with fusion-attenuating mutations indicated in red and fusion-enhancing mutations in green. The localization of the α3-helix-breaking mutations G493P, Y494P, or I495P, which abrogated TBgB´s fusogenicity in our previous study, is indicated by “pre-lock” (36). The green arrow indicates the ADgB-Y275 and VRgB-G585 polymorphisms, while the red arrows highlight the newly identified variations associated with altered fusogenicity; note the offset in numbering for TB (in green) and UK (in purple). (**b and c**) DSP-assay for quantification of cell-cell fusion activity mediated by UKgB-ΔC or TBgB-ΔC or point mutants thereof. 293T-DSP-mix cells were transfected with plasmids encoding the indicated gB variants or an empty vector (mock). Renilla luciferase activity was measured 24 h post-transfection by luciferase reporter assay and expressed as RLU/s. Experiments were performed three times (three dots); columns depict the mean of biological triplicates ± standard deviations. Statistical analysis was performed by using one-way ANOVA using Bonferroni’s multiple comparison test, **: *P* < 0.01, ****: *P* < 0.0001. *P*-values refer to cells transfected with UKgB-ΔC (**b**) or TBgB-ΔC (**c**), respectively, and were not statistically significant unless indicated.

Swapping UK-DomI into fusion-competent VR led to severely reduced fusion activity (Fig. 3b), and substituting D274N in the UK-DomI/TBgB-chimera only partially increased autonomous fusion activity (Fig. 3c). These results suggested that certain UK-restricted polymorphisms interfere with fusogenicity (Fig. 5a). In fact, substituting Dom I S217 with leucine—encoded by VR and all other gB3 members—enhanced the autonomous fusion activity of UKgB-ΔC by approximately 145-fold, although it did not reach the 340-fold increase observed with D274N (Fig. 5b). In contrast, K363E in Dom II did not significantly affect UK´s fusion activity. Beyond those polymorphisms, Dom III caught our particular attention, as we recently demonstrated that the α3-helix-breaking mutations G493P, Y494P, I495P, or C507S abrogated the fusion activity of TBgB/VSV-G (36). Likewise, substitution of N493S did not render UKgB-ΔC fusion-competent. Mutation of 566TIKDST/NVKESP571 in Dom IV resulted in an approximately 143-fold increase in fusion activity, surpassing the effect of S587G (Fig. 5b). Conversely, substituting H589Y alone or in combination with T583N, or transferring the VR-encoded F613, did not confer fusion activity to UK. Thus, it appears that strains belonging to gB5 have evolved the two fusion-attenuating mutations—S217 in Dom I and 566TIKDST571 in Dom IV—which independently account for the lack of autonomous membrane fusion by UKgB-ΔC.

To confirm these polymorphisms as regulators of fusion, they were tested in the backbone of the fusion-competent TBgB-ΔC. Consistent with the previous findings, the introduction of L215 resulted in a 2-fold increase, whereas the S215 encoded by UK reduced TB’s fusion activity by 20% (Fig. 5c). Similarly, individual substitutions 566NV/TI567 or E569D decreased fusion activity by about 20%, whereas P571T caused a reduction of approximately 50%. These results suggest that the functional contribution of the Dom IV region 566TIKDST/NVKESP571 arises from the combined effect of multiple residues, with position 571 playing a particularly important role. Additionally, TB’s residue 215 is crucial in fusion regulation, with leucine acting as a potent enhancer of gB-ΔC-mediated fusion, while serine acts inhibitory.

### Mapping determinants of autonomous fusion regulation within structural Dom I

**Fig 6:**
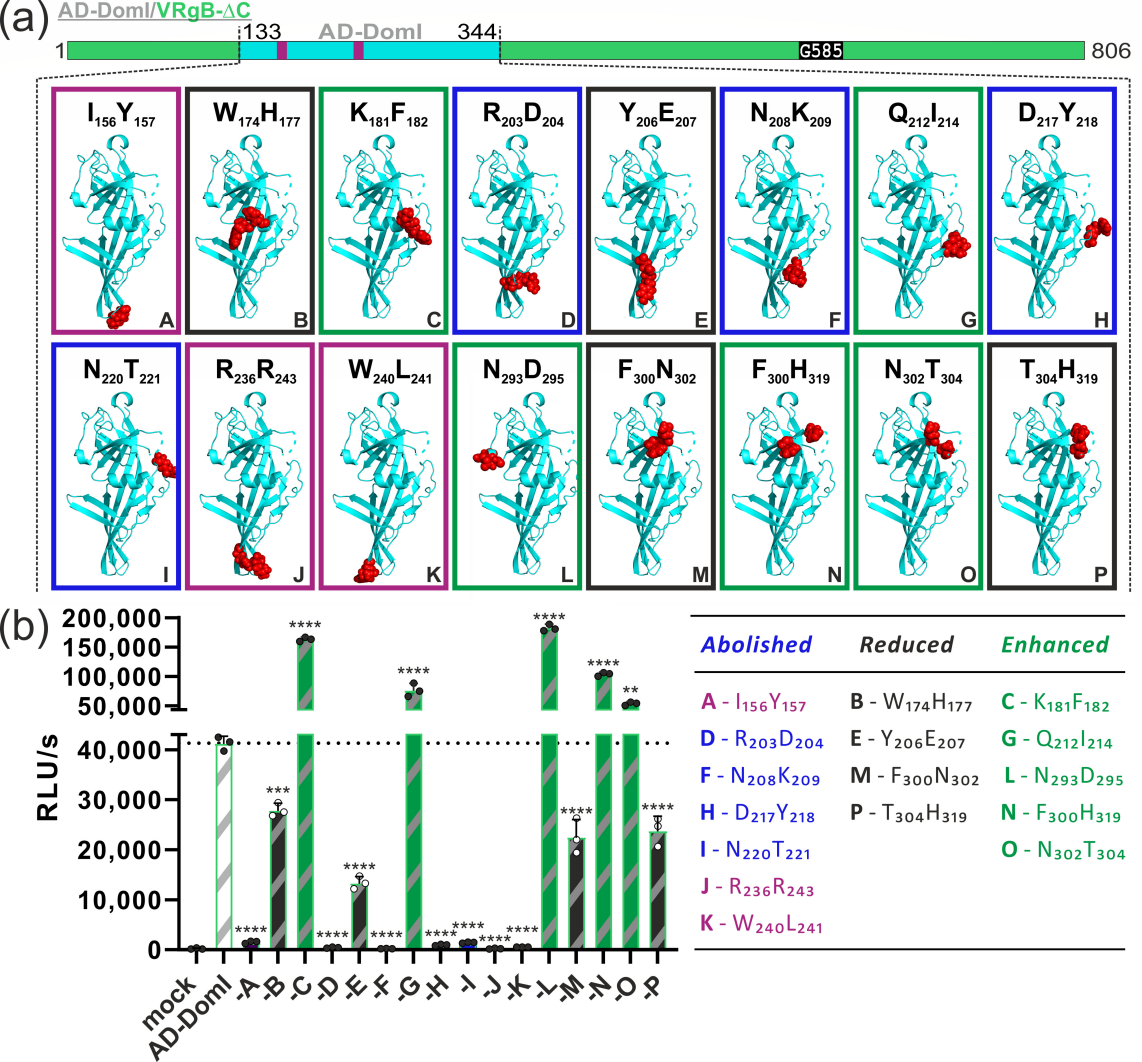
Mapping of residues in Dom I that regulate gB’s fusion activity. (**a**) Schematic representation of ADgB-DomI and mutants thereof inserted into the backbone of VRgB-ΔC. Surface-exposed amino acids that were exchanged for alanine are indicated in red space-filling representation within the crystal structure of ADgB-DomI (gB2, from PDB: 5CXF [19]). (**b**) DSP-cell-cell fusion assay of 293T-DSP-mix cells that were transfected with plasmids encoding the indicated mutants before Renilla luciferase activity was measured at 24 h post-transfection. Experiments were performed three times (three dots); columns depict the mean of biological triplicates ± standard deviations. Statistical analysis was performed by using one-way ANOVA using Bonferroni’s multiple comparison test, **: *P* < 0.01, ***: *P* < 0.001, ****: *P* < 0.0001. *P*-values refer to cells transfected with AD-DomI/VRgB-ΔC. (Table) Mutants were grouped based on their effect on fusogenicity: green indicates enhanced fusion, while blue, purple, and black indicate abolished or reduced fusion activity, respectively. Purple indicates substitutions located within or adjacent to the fusion loops.

Having established the Dom I polymorphisms L/P/S215, D272N, and D275Y as critical regulators of fusion in the absence of gH/gL, we sought to determine whether this domain contains additional elements that influence fusion regulation. We took advantage of an existing panel of AD-Dom I mutants, in which two closely situated surface-exposed residues were replaced with alanine (Fig. 6a) (35). These mutants were transferred into the backbone of fusion-competent AD-DomI/VRgB-ΔC (compare Fig. 3b). DSP-cell-cell fusion assays revealed that seven substitutions, A (I156A + Y157A), D (R203A + D204A), F (N208A + K209A), H (D217A + Y218A), I (N220A + T221A), J (R236A + R243A), and K (W240A + L241A), strongly reduced or abolished fusion (Fig. 6b). In fact, mutants A, J, and K are located within or near the fusion loops, which are essential for membrane fusion, as previously demonstrated by the critical roles of residues Y155/I156/Y157 and W240 (17, 37). Mutants D and F, as well as H and I, are situated in two opposing loops that appear to form interfaces possibly stabilizing the prefusion structure and/or coordinating conformational rearrangements during the fusion process. Consistent with this assumption, these sites are 100% conserved across the 475 publicly available gB sequences, underscoring their functional importance. A second set of substitutions, including B (W174A + H177A), E (Y206A + E207A), M (F300A + N302A), and P (T304A + H319A), reduced fusion activity by 32%–67%, suggesting that these positions are involved in regulating gB’s fusion activity in the absence of gH/gL. In contrast, the mutants G (Q212A + I214A) and O (N302A + T304A) increased fusion by more than 30%, while constructs C (K181A + F182A), L (N293A + D295A), and N (F300A + H319A) increased RLuc signals by more than 150% compared to the parental AD-DomI/VRgB-ΔC control, indicating that the native residues at these positions generally constrain fusogenicity (Fig. 6b). Collectively, di-alanine scanning revealed three functional classes within Dom I: (i) essential sites, whose perturbation impairs fusion; (ii) sites that reduce fusion; and (iii) autoinhibitory sites, where alanine substitution enhances fusion—in summary underscoring Dom I’s vital role in regulating membrane fusion by C-terminally truncated gB in the absence of gH/gL.

### Dom I–IV interfaces serve as key regulators of gB-ΔC-mediated fusion activity

**Fig 7:**
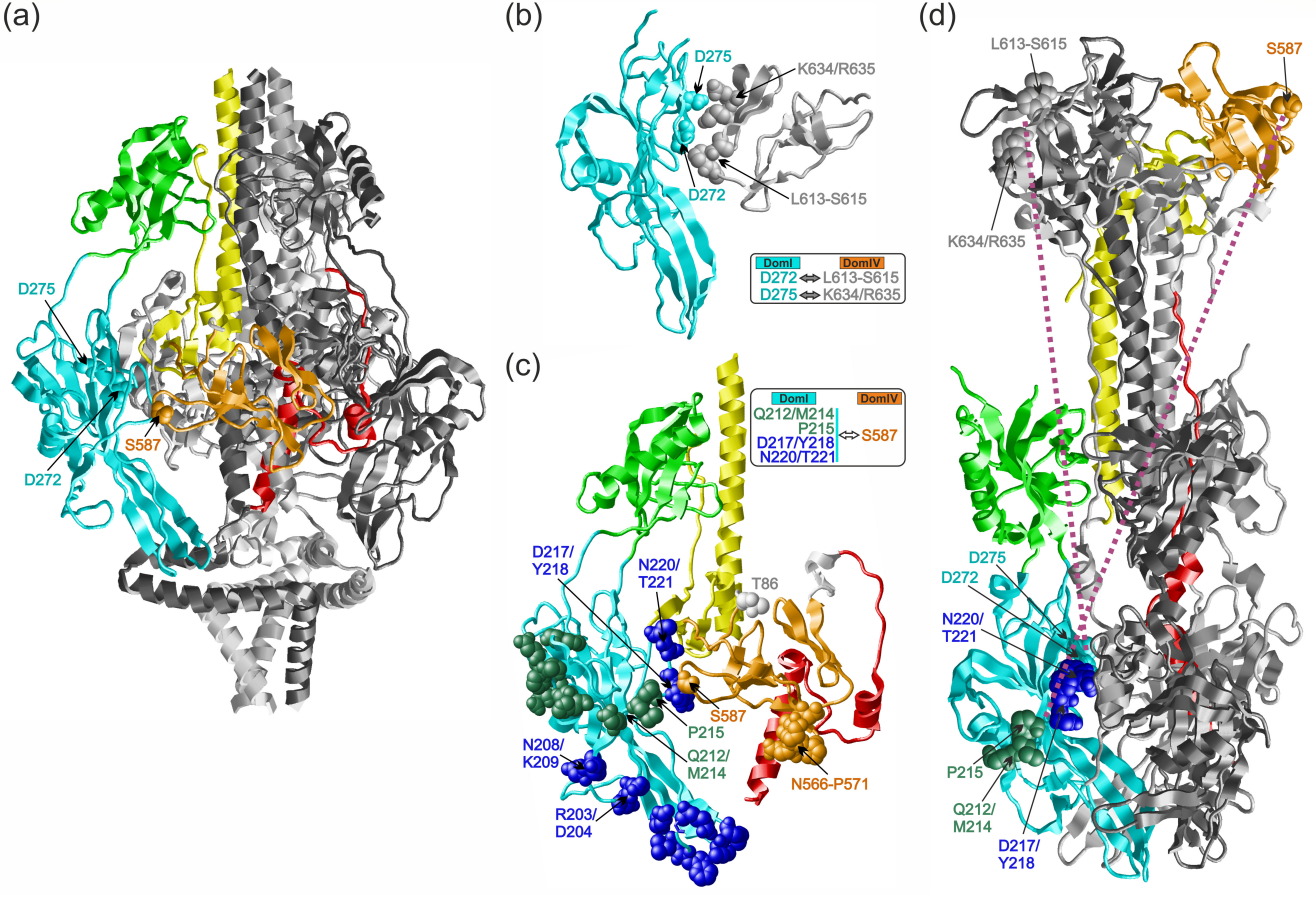
Structural insights into the role of Dom I and Dom IV in regulating HCMV gB fusogenicity. (**a–c**) Structure of the trimeric prefusion Towne-gB (gB1, PDB: 7KDP (18)) with one subunit colored according to Fig. 1 and the other two protomers in light or dark gray. The key sites for the control of fusogenicity of three different gB-genotypes are shown in space-filled presentation: D272, D275, and S587. (**b**) Interactions of D272 and D275 located in Dom I with two sequence patches (L613-S615 and K634/R635) of Dom IV. Dom I and Dom IV belong to different subunits of the trimer and are colored cyan and gray, respectively. (**c**) Fusion-activating (dark green) or fusion-blocking positions (blue) identified in Fig. 6 are indicated as spheres. The Dom IV residue S587 in gB1/gB5 (S586 in gB2; S585 in gB3/gB4) interacts intramolecularly with the sequence patch D217-T221 of Dom I from the same subunit of the trimer. Additionally indicated are the critical positions Q212/M214, P215, and 566NVKESP571 (N566-P571) of Towne-gB, which are substituted for I214 in AD169 (compare with Fig. 3 and 6a) or S217 and 566TIKDST571 in strains belonging to non-fusogenic gB5 (Fig. 5a). The heterogeneous N-terminus of gB (Fig. 2) is not resolved in the structure, which starts at T86. (**d**) Structure of the trimeric postfusion gB (PDB: 7KDD (18)); same color coding as in panel (**a**. The purple dotted lines connect pairs of residues that were directly interacting in the prefusion conformation, indicating the complete loss of the respective contacts.

A visualization of the residues that critically affected the fusogenicity reveals that they cluster in distinct regions of the Towne-gB (gB1) prefusion structure (Fig. 7). Most of the fusion-enhancing mutations are situated at the outer layer of Dom I, which might indicate a region involved in recruitment of a cellular interaction partner, given that viral factors such gH/gL-containing complexes are absent in our experimental setup. Unlike Towne-gB D272 and D275, which interact with Dom IV of a neighboring protomer in the gB-homotrimer, S587 in Dom IV engages within the same subunit (Fig. 7a through c). The latter interface includes the hyperfusogenic mutant Q212A + I214A, the L/P/S215 polymorphism, and the fusion-abrogating mutants D217A + Y218A and N220A + T221A (Fig. 7c). Regardless of whether these interactions are intra- or inter-subunit, the Dom I–IV contacts are lost in the postfusion conformation (Fig. 7d). This suggests that the strength of these intra- and inter-protomer interactions between Dom I and Dom IV in the prefusion conformation of gB influences the kinetics of the pre- to post-fusion transition, thereby affecting fusogenicity. Notably, gB5 incorporates an additional, unique layer of regulation provided by the loop formed by the Dom IV region 566TIKDST571, which replaces the 566NVKESP571 loop of Towne-gB (Fig. 7c).

### Critical interplay of Dom I and Dom V and impact of polymorphisms from clinical isolates

**Fig 8:**
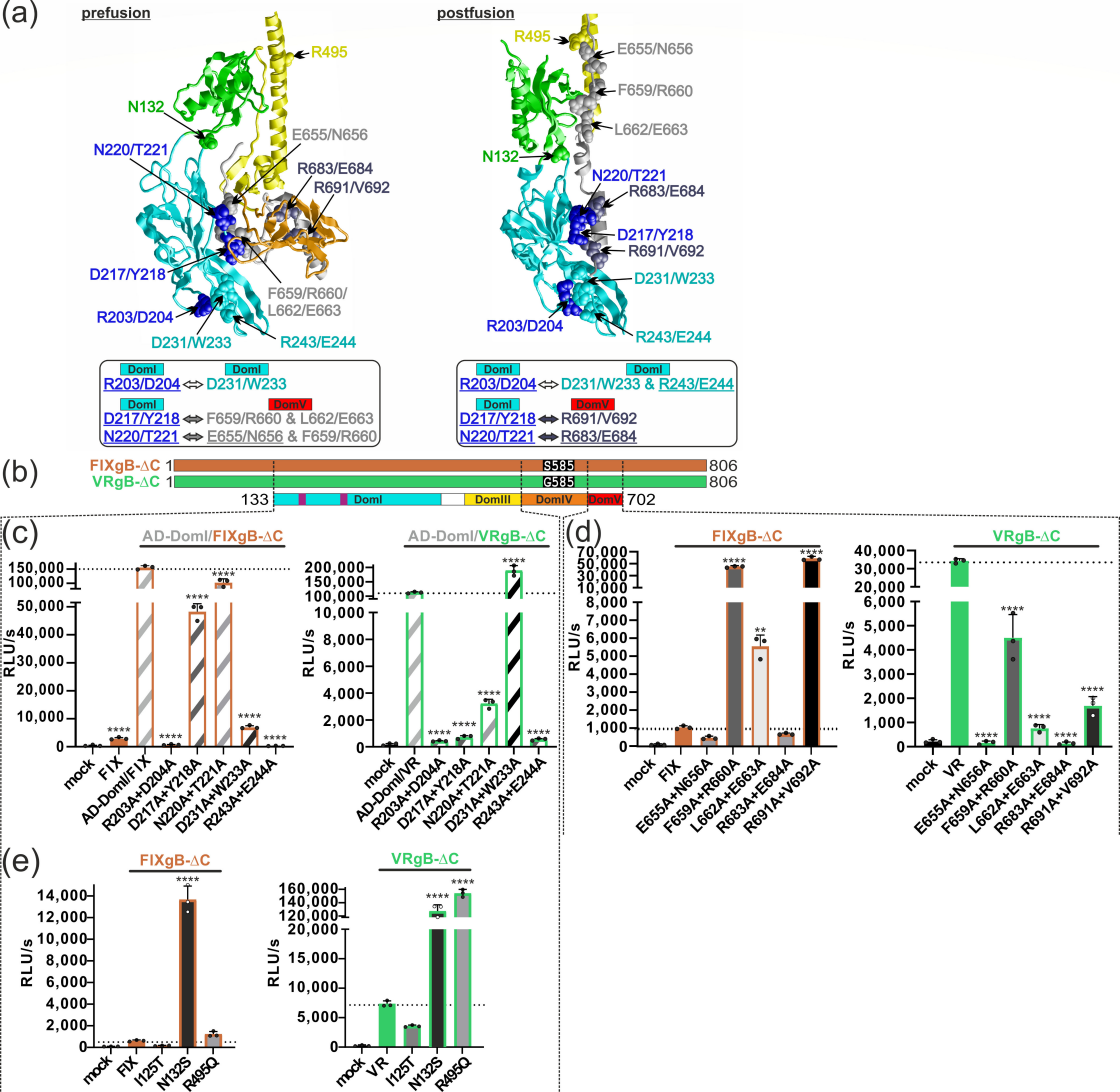
Mapping of critical residues in Dom I and Dom V regulating gB-mediated cell-cell fusion. (**a**) The fusion-abrogating residues R203/D204, D217/Y218, and N220/T221—corresponding to mutants D, H, and I in Fig. 6b and 7c—are indicated as blue spheres within the pre- and post-fusion conformation of Towne-gB (gB1, PDB: 7KDP and 7KDD (18)). The isolate polymorphisms N132 and R495 from (**e**) are indicated as green or yellow spheres, respectively. Of note, the indicated residues and positions refer to the gB3 strains VR and FIX, which were analyzed in this experiment. The available structures reveal that Dom I forms (**i**) an intramolecular self-association interface and (ii) an intermolecular interface with Dom V of a neighboring subunit in the gB trimer; these are shown with open and filled double arrows, respectively. Prefusion-stabilizing residues are visualized as gray spheres, while blue-gray spheres indicate postfusion-stabilizing residues. Underlined residues were identified as crucial for fusion regulation (see panels c and d). (**b**) Schematic representation of FIXgB-ΔC and VRgB-ΔC used for mutational analysis. (**c and d**) Cell-cell fusion assessment of Dom I (**c**), Dom V mutants (**d**) or polymorphisms from clinical isolates (**e**) using the DSP assay. Experiments were performed three times (three dots); columns depict the mean of biological triplicates ± standard deviations. Statistical analysis was performed by using one-way ANOVA using Bonferroni´s multiple comparison test, **: *P* < 0.01, ****: *P* < 0.0001. *P*-values refer to the respective parental strain, which is highlighted by a dotted line and were not statistically significant unless indicated.

To further dissect the intra- and inter-protomer interactions within gB, we analyzed the potential interfaces formed by the DomI-mutants D, F, H, and I (Fig. 6b). Mutant F, which has N208 + K209 exchanged for alanine, lacks an interaction interface and likely lost its fusion activity due to improper protein processing caused by the absence of the known N-glycosylation site at N208 (19). Putative contact sites of D, H, and I (Fig. 8a) were substituted for alanine before assessing the cell-cell fusion capacity. Consistent with the results above, all mutants abrogated fusion of AD-DomI/VRgB-ΔC (Fig. 6b and 8c). In FIX, the impact of mutants H and I (i.e., D217A + Y218A and N220A + T221A) was less pronounced, reducing fusion by 69% and 34%, respectively, but not entirely inactivating the protein (Fig. 8c). Mutant D (R203A + D204A) may form intraprotomer interactions with D231/W233 in the prefusion conformation and/or D231/W233 and R243/E244 in the postfusion conformation. In fact, mutant D231A + W233A abrogated cell-cell fusion in FIX but increased the activity of VR by more than 67%. In contrast, R243A + E244A abrogated fusion in both backbones (Fig. 8c), and R243A was also contained within the fusion-defective mutant J (R236A + R243A) (Fig. 6b). Collectively, the Dom I residues R203/D204, R236, and R243/E244 are universally required for intraprotomer interactions to regulate the fusion activity of gB, either by contributing to the hydrophobic fusion loops and/or stabilizing the pre- or post-fusion conformation. In contrast, D231/W233 demonstrate a backbone-dependent impact on fusion regulation.

A detailed inspection of the interface formed by Dom I D217-T221 (corresponding to mutants H and I) suggests that the well-conserved Dom V of the neighboring gB-protomer might additionally be involved in stabilization (Fig. 8a). In fact, loosening the potential prefusion interface of D217/Y218 in the double mutants F659A + R660A or L662A + E663A enhanced fusion activity of FIX by more than 40-fold or 5-fold, respectively (Fig. 8d). Similarly, disruption of the postfusion interface in mutant R691A + V692A enhanced fusion of FIX by more than 50-fold. Conversely, all tested Dom V mutants significantly decreased activity of fusion-competent VR, indicating that this domain operates in a backbone-dependent regulatory mode (Fig. 8d). Disrupting the N220/T221 prefusion interface with E655A + N656A or the postfusion interface with R683A + E684A entirely abolished fusion, highlighting the crucial interprotomer interactions between Dom I and Dom V in fusion regulation.

Notably, the critical fusion-enhancing polymorphisms known so far have all been identified in well-established laboratory strains or their derived bacterial artificial chromosomes; however, these substitutions are rarely found in clinical isolates. Encouraged by the findings of this present study, we searched for additional relevant polymorphisms in newly obtained gB sequences. We draw our attention to the gB3 urine isolate “C10_5,” encoding the so far uncharacterized polymorphisms I125T and Y492N, which are absent in any annotated gB sequence (Irene Görzer). We also included the adjacent N132S and R495Q identified by direct sequencing of the gB3 strains “HAN-SCT19” (QZX45917) or “HANRTR8” (AQN71866), respectively. While substitution I125T reduced fusion by 2-fold, the mutation N132S led to a more than 17-fold increase of fusion in both backbones (Fig. 8e). Conversely, the R495Q polymorphism increased fusogenicity by 2-fold to 20-fold when transferred to FIX or VR, respectively, thereby reconfirming the critical role of the Dom III α3-helix as a fusion-regulating determinant (36). Furthermore, in Dom I, we have identified previously uncharacterized polymorphisms, which significantly impact the gH/gL-independent fusion activity of gB: I125T diminished fusion, whereas N132S enhanced it. In summary, our results show that Dom I is intricately connected to Dom IV and Dom V through a finely tuned inter-domain communication network. Within this network, subtle sequence polymorphisms alter long-range interactions among the domains, thereby modulating the overall fusogenic activity of C-terminally truncated gB.

## DISCUSSION

HCMV’s membrane fusion is mediated by the structurally conserved gB, which serves as a key target for antiviral strategies (8, 12, 38–40). Accumulated mutations and recombinations led to multiple gB-genotypes, with significant implications for immunity and pathogenicity (23, 24, 26, 41, 42). Our integrative approach, combining *in silico* analysis with structural and functional dissection, demonstrates that the default state of full-length gB and gB-ΔC is fusion-inactive, as evidenced by the typical gB1–gB5 representatives: TB-N272D, AD-Y275D, FIX, C194, and UK. It can be assumed that these proteins fold into a low-energy postfusion conformation when expressed without other viral proteins (16, 17). Previous studies attributed the hyperfusogenic phenotypes of AD and VR to D275Y and S585G, respectively, which both accelerate viral entry and promote cell-cell fusion when expressed in the context of the whole virus (27, 28, 43). Likewise, the TB40/EE substrain infects epithelial cells more efficiently and induces larger multinucleated syncytia in both epithelial and fibroblast cultures than parental TB40/E, an effect attributed to substitutions in IE2 and in UL128, UL130, and UL141 (components of the gH/gL or gH/UL116 complexes) (44–48). In our DSP assay, the syncytium-forming activity of C-terminally truncated gB, assessed in the absence of other viral proteins, closely mirrored the phenotypes observed during infection with the corresponding parental viruses. This concordance suggests that the mechanisms driving gB ectodomain reorganization are shared between gH/gL-dependent and -independent fusion, supporting the use of gB-ΔC as a tractable model for elucidating general principles of gB fusion regulation.

Building on this knowledge, we have now identified D272N as an additional fusion-accelerating polymorphism. Notably, while all published gB1 strains, including Merlin and Towne, encode a D272 akin to gB2–gB5, the N272 variant is found exclusively in a restricted subset of gB1 strains such as the original TB40/E isolate, along with the closely related variants TB40/EE (QTF98520) and TB40/EF (QTF98353), but also in clinical cCMV isolate “S5_2-49-mid2-ul55” (WMN01606) and urine isolate “P10” (QPZ45000) (34, 45, 49). Likewise, VR’s S585G has so far been found exclusively in the gB3 isolates “P4, P14, and P15,” which were sequenced from congenitally infected low-passaged clinical isolates due to their “aggressive” syncytium-forming phenotypes (43, 49, 50). Notably, N272 and G585 enhance fusogenicity across all tested genotypes, with G585 having a more pronounced effect. Their lack of additive fusogenicity suggests that each site independently confers fusion competence by either (i) stabilizing the metastable gB ectodomain in its prefusion conformation or (ii) loosening the clamp that maintains gB in a fusion-inactive state. The gB3 isolates “HAN-SCT19” (QZX45917) and “HANRTR8” (AQN71866), sequenced from clinical material, encode unique hyperfusogenic substitutions at distinct sites: N132S at the border of Dom I, and R495Q in the central α3-helix of Dom III. It is tempting to speculate that these substitutions disrupt the relative localization of gB domains, rendering the protein fusion-competent; however, this warrants experimental validation. The detection of fusion-enhancing polymorphisms in clinical isolates suggests that these are naturally occurring rather than cell culture artifacts, but it remains to be determined whether they correlate with disease severity and/or virus transmission.

Mechanistically, TB’s N272 and AD’s Y275 disrupt Dom I interprotomer interactions with Dom IV at L613–S615 and K634/R635, respectively. Alternatively, VR evolved G585 in Dom IV, thereby disrupting its intraprotomer interface with Dom I, composed of Q212/M214, L/P/S215, D217/218, and N220/T221. This interface is further stabilized by Dom I interactions with itself, as di-alanine substitution of R203/D204 or its contact site R243/E244 abrogated fusion, likely by preventing postfusion formation. The D231A + W233A mutation, which interferes with both pre- and post-fusion contacts, abolished fusion in AD-DomI/FIX but increased activity in AD-DomI/VR. This likely reflects VR’s advanced position in the fusion trajectory due to G585, making it less susceptible to inhibition and enhancing fusion by preventing the postfusion conformation. The critical Dom I–IV interface is further stabilized through Dom I’s interprotomer contacts with Dom V. For this, E655/N656 and R683/E684 are of particular importance, as their substitution with alanine abrogated fusion in every construct. Conversely, F659A + R660A or L662A + E663A conferred fusion competence to FIX, likely by loosening its prefusion interface with D217/Y218 and/or N220/T221. In contrast, R691A + V692A enhanced FIX by preventing postfusion stabilization via D217/Y218 and/or disrupting the prefusion alpha-helical bundle. Collectively, these findings mechanistically explain how Dom V acts as a crucial regulator of fusion for C-terminally truncated gB, as supported by our previous publication (36).

The loop neighboring G585 in Dom IV is also involved in fusion regulation: the gB5 sequence 556TIKDST571 acts inhibitory, while the nearly 100% conserved 566NVKESP571 of all other genotypes permits fusion. However, attenuating substitutions like E569D occasionally occur in strains such as TR [AGL96655], isolated from an ocular swab from an AIDS patient (51, 52), as well as in “NL/Rot2/Urine/2012” (AMJ52758), “LUS243” (QBF76487) from breast milk, and “SYD-SCT2” (QIA45372) from plasma. The relative positioning of critical fusion regulators, such as Dom I Q212–T221, near the Dom IV loops containing G585 and 566NVKESP571, underscores their crucial intraprotomer communication. The structurally unresolved N-terminus, which transitions into Dom IV, may overlap this critical interface. In fact, in the context of C-terminally truncated gB, the NTD was identified as a major contributor to fusion regulation, exhibiting backbone-dependent effects when transferred between genotypes.

Our study further revealed critical Dom I residues (K181/F182, Q212/I214, N293/D295, F300/H319, and N302/T304), which, when replaced by alanine, enhanced fusion of gB-ΔC. Residues 181KFA183 are variably substituted by R/SHS, and residue 214 is predominantly methionine (M214), the I214 variant—present in AD169—appears in only about 9% of all strains. All other sites are 100% conserved in published clinical isolates, underscoring their role in maintaining the structural integrity of the prefusion trimer. Located on the solvent-exposed outer surface of Dom I, these residues may, in the absence of other viral proteins, recruit cellular interaction partners and/or stabilizing intermediate fusion states. Notably, Q212/I214 are in close proximity to the L/P/S215 polymorphism. L215 is also found in gB1-isolates, such as the congenital CMV isolates “PRA1” (AQN69676.1), or “PRO-66-mid86-ul55,” “PRO-66-mid84-ul55,” and “PRO-66-mid82-ul55” (34), while Q212R was found in “PRO-66-mid81-ul55” (WMN01601), ultimately underlining the *in vivo* relevance of these polymorphisms. Based on our findings, it is tempting to suggest that these substitutions affect the syncytium-forming capacity and therefore also the pathogenicity and transmission of the respective isolates.

As an immunodominant envelope antigen, gB elicits strong B-cell and T-cell responses (39, 53). However, vaccines such as the primarily monomeric Towne-gB ectodomain + CTD (gB/MF59) (54–57), prefusion-stabilized Towne-gB ectodomain (gB-C7) (58, 59), and mRNA-encoded full-length Merlin-gB with pentamer (mRNA-1674 and NCT05085366) (60–63) provided only partial protection; notably, both Towne and Merlin are gB1 strains that encode D272. Our findings may, among other factors, help explain the limited success of these approaches via two non-exclusive mechanisms: disruption of the interdomain regulatory network may have compromised antigenic integrity, and low expression of the pentamer receptors NRP2 or OR14I1 in skeletal muscles—the site of mRNA-1647 administration—may have impeded gB activation (64–66). This raises the possibility that utilizing fusion-enhancing substitutions like N272, Y275, or G585 could unmask normally occluded antigenic structures and thereby improve immunogenicity; we are currently testing this hypothesis. Such an approach is supported by findings that the gB/MF59 antigen induced rare anti-AD-6 responses, effectively blocking cell-associated virus spread across herpesvirus subfamilies (38, 67).

This study provides the first mechanistic explanation of how gB polymorphisms drive distinct fusion activities. The widespread distribution of these polymorphisms across clinical HCMV isolates highlights their continuous emergence and potential to evolve *in vivo*. When considering the additional risk of intrahost recombination, there is a critical need for diagnostics targeting the full-length gB coding sequence. Our experimental approach offers an effective screening tool for identifying key polymorphisms, which is essential for understanding how gB variants may affect transmissibility, pathogenicity, and immune responses, and for optimizing vaccine design.

## MATERIALS AND METHODS

### Cell culture

The human embryonic kidney HEK293T-DSP-mix cells, consisting of HEK293T cells stably expressing the dual split proteins DSP1-7 and DSP8-11 co-cultured in a 1:1 ratio (32)), were cultured in Dulbecco’s modified Eagle medium (Gibco, Thermo Fisher Scientific, Darmstadt, Germany). The medium was supplemented with 10% fetal calf serum (Merck, Sigma-Aldrich, Taufkirchen, Germany), glutamine (100 μg/mL), and gentamicin (350 μg/mL).

### Oligonucleotides and expression constructs

Primers used for site-directed mutagenesis were purchased from biomers.net GmbH (Ulm, Germany) and are listed in Table 1.

**TABLE 1:** Primers used for cloning or site-directed mutagenesis

Primer no.	Name	Sequence
0-84	3Xho-gB807-STOP	gcatctcgagtcacagggaggtatccttggtgctgc
4-90	5gB D272N + Y275D	cgccaccagcaccggcaacgtggtggacatcagccccttctac
4-91	3gB D272N + Y275D	gtagaaggggctgatgtccaccacgttgccggtgctggtggcg
4-92	5TBgB N272D	gccaccagcaccggcgacgtggtggacatcag
4-93	3TBgB N272D	ctgatgtccaccacgtcgccggtgctggtggc
5-09	5PpuM1,EcoN1gB-DomI	gcataggaccttgacgagggcatcatggtsgtgtacaagmgraacatcgtggcccacacc
5-10	3Pfo1-gB	gcattcctggatgtcccagctgatcacgctgtcgg
5-11	5gB S585G	gatcttcaacttcgtgaacggcagctacgtgcagtacgg
5-12	3gB S585G	ccgtactgcacgtagctgccgttcacgaagttgaagatc
6-13	5VRgB E655A + N656A	gatatcgaccccctggcggccaccgacttccgagtgctgg
6-14	3VRgB E655A + N656A	cagggcgatcatgctgtccacggtgc
6-15	5VRgB F659A + R660A	ctggagaacaccgacgccgcagtgctggagctgtacagc
6-16	3VRgB F659A + R660A	ggggtcgatatccagggcgatcatg
6-17	5VRgB L662A + E663A	gacttccgagtggcggcgctgtacagccagaagg
6-18	3VRgB L662A + E663A	ggtgttctccagggggtcgatatccagg
6-19	5VRgB R691A + V692A	cagctacaagcaggccgcgaagtacgtggaggac
6-20	3VRgB R691A + V692A	gcatgatctcctccaggtcgaacacg
6-21	5VRgB R683A + E684A	ggaggagatcatggcggcattcaacagctacaagc
6-22	3VRgB R683A + E684A	aacacgttgctgctcctcagctcc
6-25	5VRgB D231A + W233A	cgtgaccgtgaaggccaggcgcactctcggggcagc
6-26	3VRgB D231A + W233A	tatctggtgctgtgggtg
6-27	5VRgB R243A + E244A	gcacctggctgtacgcagcgacatgcaacctgaactg
6-28	3VRgB R243A + E244A	tgccccgagagtgccactg
6-32	5UKgB K363E	catcaggagcgaggccgaggacagctaccacttcagc
6-33	3UKgB K363E	gttctctcgctggcctccca
6-34	5UKgB N493S	tacgacaccctgcggagctacatcaacagagcc
6-35	3UKgB N493S	ggtgaactgcagctgagcgta
6-36	5UKgB H589Y	cgccaacagctcttacgtgcagtacggacagc
6-37	3UKgB H589Y	aaggtgaagatcacgac
6-38	5UKgB L613F	ccgaggagtgtcagttccctagcctgaagatc
6-39	3UKgB L613F	ttctgtggttgcccagcaggatc
6-58	5ADgB Y275D	ggcgacgtggtggatatcagccccttct
6-59	3ADgB Y275D	ggtgctggtggcgaag
6-88	5Bam-C194 + UKgB aa1	gcatggatccatggagagccgcatttggtg
6-89	3OE-C194 + UKgB aa124	caccatgatgccctcgtcaaggtcctcattgatag
6-90	5OE-TBgB aa124	ctatcaatgaggaccttgacgagggcatcatggtg
6-93	5UKgB T583N	gtcgtgatcttcaatcccgccaacag
6-94	3UKgB T583N	gggcctagagtaacag
6-95	5UKgB 566TIKDST/NVKESP571	ctgcggatatgaacgtcaaggagagccccggccgctgttac
6-96	3UKgB 566TIKDST/NVKESP571	caccttcacgctggtc
7-11	5UKgB S217L	accatgcagctgatgctcgacgactacagcaa
7-12	5UKgB S217L	cttgttctcgtagctgtccc
7-35	5TBgB P215L	ccatgcagctgatgctcgacgactacagcaatacc
7-36	3TBgB P215L	tcttgttctcgtagctg
7-37	5TBgB P215S	gaccatgcagctgatgtccgacgactacag
7-38	3TBgB P215S	ttgttctcgtagctgtc
7-39	5TBgB 566NV/TI567	gtgctgcgggacatgaccatcaaagaaagccccgg
7-40	3TBgB 566NV/TI567	tttcacgctggtctgattg
7-41	5TBgB E569D	gacatgaacgtgaaagacagccccggcagatg
7-42	3TBgB E569D	ccgcagcactttcacgctggtc
7-43	5TBgB P571T	catgaacgtgaaagaaagcaccggcagatgctacagcc
7-44	3TBgB P571T	tcccgcagcactttcacgc
7-45	5UKgB Q51R	caagcgccgccagatctcggagcggaagcgtgtctcac
7-46	3UKgB Q51R	ttgtgtggctgctgtggtgg
7-47	5UKgB 57HH/QR58	gagcggaagcgtgtctcagcgcgtgacaagcagccag
7-48	3UKgB 57HH/QR58	tgagatctggcggcgcttgttg
7-49	5UKgB D68G	ccagaccgtgtctcacggcgtgaacgagaccatc
7-50	3UKgB D68G	ctgcttgtcacgtggtgagac
7-55	5FIXgB I125T	ggacctggacgagggcaccatggtcgtgtacaagcgg
7-56	3FIXgB I125T	tcgttgatgggcttcat
7-57	5FIXgB Y492D	cgacaccctgaggagcgacatcaaccgggctctggc
7-58	3FIXgB Y492D	taggtgaactgcagctggg
7-59	5FIXgB N132S	ggtcgtgtacaagcggagcatcgtggcccacaccttcaag
7-60	3FIXgB N132S	atgatgccctcgtccaggtcc
7-61	5FIXgB R495Q	ctgaggagctacatcaaccaggctctggcccagatcgc
7-62	3FIXgB R495Q	ggtgtcgtaggtgaactgcag
7-73	5TBgB R49Q	tgccgcccactctcagagcggcagcgtgtcac
7-74	5TBgB R49G	tgccgcccactctgggagcggcagcgtgtcac
7-75	5TBgB S50F	tgccgcccactctcggttcggcagcgtgtcacag
7-76	5TBgB G51S	tgccgcccactctcggagcagcagcgtgtcacagagagtg
7-77	3TBgB R49/S50/G51	gatgttgttctgctgctg
7-78	5TBgB S59F	gtcacagagagtgaccttcagccagaccgtgtcccacg
7-79	3TBgB S59F	acgctgccgctccgag
7-80	5TBgB H65Y	cagccagaccgtgtcctacggcgtgaacgagacaatc
7-81	3TBgB H65Y	ctggtcactctctgtgac
7-86	5TBgB 36SH-NGS39	caccagcgccacccacaacggatctcacagcagcagaacaac
7-87	3TBgB 36SH-NGS39	cctcttgtagagctgcta
7-90	5ADgB Y53S	ccagaccagaagcgtgtccagccagcacgtgacc
7-91	3ADgB Y53S	gcgctggtggttctgctg

The expression plasmids coding for HCMV AD169gB (P06473), AD169gB-807, AD169gB/VSV-G, and TB40/EgB (ABV71586) were described previously (16, 17). The expression constructs of VR1814gB (XBA02838), FIX-BACgB (AC146907), C194AgB (AAA45925), and UKNEQAS1gB (AHJ86153) were obtained by cloning of the respective codon-optimized nucleotide sequences synthesized by Synbio (Nanjing, China). The gB-ΔC truncations and gB/VSV-G chimeras were generated through cut-and-paste cloning using the restriction enzymes BamHI and, EcoNI, AleI, or EcoRI, respectively, or EcoRI and XhoI (New England Biolabs, Ipswich, MA, USA and Thermo Fisher Scientific, Waltham, USA). AD-Nterm- and VR-Nterm/TBgB-ΔC chimeras were produced by using restriction enzymes EcoNI and EcoRI, or BamHI and EcoNI, respectively. The C194-Nterm- and UK-Nterm/TBgB-ΔC constructs were generated by overlap extension PCR. For this, the Nterm of C194 or UK was amplified using primer pairs 6-88 + 6-87 or 6-88 + 6-89, respectively, and the TBgB-ΔC fragment was amplified using primers 6-90 and 0-84, with the full inserts assembled using primers 6-88 and 0-84. DomI/VRgB-ΔC and DomI/TBgB-ΔC chimeras were produced by substituting the Dom I fragments using the restriction enzymes PpuMI and PflMI or PpuMI and PfoI, respectively (New England Biolabs, Ipswich, MA, USA). The AD-DomI-mutants were generated by PCR amplification of Dom I mutants (from Wiegers et al. (35)) using primers 5-09 and 5-10, followed by digestion with PpuMI and PflMI or PpuMI and PfoI, respectively, and ligation into TBgB-ΔC or VRgB-ΔC digested with the same enzymes. The other mutants were generated by using Vent DNA polymerase (Catalog #M0254S, New England Biolabs GmbH, Frankfurt am Main, Germany) or Q5 Site-Directed Mutagenesis Kit (Catalog #E0554S, New England Biolabs GmbH, Frankfurt am Main, Germany) with the pairs of primers listed in Table 1. The sequence integrity of all newly generated plasmids was verified by automated DNA sequencing (Macrogen, Amsterdam, the Netherlands), and the results were analyzed using Vector NTI 9.1.0 or SnapGene 7.2.1.

### Dual split protein cell membrane fusion assay (DSP assay)

Cell-cell fusion was quantified using the DSP assay, in which membrane fusion enables intracellular complementation of split Renilla luciferase (RLuc) and split GFP fragments (68, 69). Here, we employed a previously described and validated DSP assay (32), co-culturing complementary HEK293T reporter cell lines (DSP1-7 and DSP8-11) at a 1:1 ratio. These 293T-DSP-mix cells were seeded into ViewPlate-96 Black dishes (3 × 10^4^ cells/well; PerkinElmer, Waltham, MA, USA), and transfected with expression constructs using calcium phosphate precipitation. After 2 h (VRgB) or 4 h (all other gB), cells were washed with phosphate-buffered saline (PBS) and supplied with fresh medium. At 24 h (VRgB) or 36 to 50 h post-transfection (all other gB), cells were incubated with 60 µM EnduRen (Promega, Madison, WI, USA), and RLuc activity was subsequently measured 2–7 h after EnduRen addition using an Orion microplate luminometer (Berthold Technologies, Bad Wildbach, Germany). Experiments were repeated independently at least three times (separate transfections on different days); the columns depict the mean of biological triplicates ± standard deviations.

### Indirect immunofluorescence analysis

For the performance of indirect immunofluorescence analysis, 293T-DSP-mix cells were cultured on coverslips and transfected with plasmid vectors or gB-variant expression constructs via calcium phosphate precipitation. Two days post-transfection, cells were washed twice with PBS and fixed in 4% paraformaldehyde solution at room temperature for 10 min. After fixation, cells were washed three times with PBS and permeabilized with 0.2% Triton X-100 in PBS for 20 min on ice. After washing four additional times, cells were blocked in PBS containing 1% bovine serum albumin (BSA) for 30 min at room temperature. Subsequently, cells were incubated with the primary antibody anti-gB 27-287, followed by Alexa Fluor 555-conjugated secondary antibody incubation for 45 min at 37°C. Coverslips were finally mounted onto microscope slides using Vectashield mounting medium containing DAPI (4′,6-diamidino-2-phenylindole; Vector Laboratories, Newark, CA, USA). Samples were analyzed using a Leica TCS SP5 confocal microscope equipped with 488 nm and 543 nm laser lines, each channel scanned separately to prevent channel overlap. Captured images were processed using Adobe Photoshop Elements 15 and compiled using CorelDraw X6.

### Cell-based ELISA (cELISA)

The cell surface expression of gB was analyzed by a cell-based ELISA (cELISA). For this, 293T-DSP-mix cells (3 × 10^4^ cells/well) were seeded in triplicates in 96-well plates coated with Poly-D-Lysine according to the manufacturer’s protocol (Gibco, Thermo Fisher Scientific, Darmstadt, Germany). Three hours post-transfection, cells were washed with DPBS and supplied with fresh medium. After 24 h, cells were rinsed once with DPBS and incubated for 30 min at 37°C with 10 µg/mL anti-gB mouse monoclonal antibody 27-287 diluted in 3% BSA/PBS. Cells were then washed five times with PBS and fixed for 10 min in 2% formaldehyde/0.2% glutaraldehyde (Merck, Sigma-Aldrich, Taufkirchen, Germany) in PBS. Following three washes with 3% BSA/PBS, cells were incubated for 30 min at 37°C with biotinylated goat anti-mouse IgG (Merck, Sigma-Aldrich, Taufkirchen, Germany) diluted 1:500 in 3% BSA/PBS. After five washes with 3% BSA/PBS, streptavidin–horseradish peroxidase (1:10,000 in 3% BSA/PBS; Cytiva, Amersham Biosciences Europe GmbH, Freiburg, Germany) was applied for 30 min. Plates were washed five times with PBS containing 0.1% Tween (Thermo Fisher Scientific, Darmstadt, Germany), then 100 µL tetramethylbenzidine (TMB) substrate, diluted 1:1 in peroxidase substrate solution B (KPL; SeraCare Life Sciences Inc., Milford, MA, USA), was added for 10 min. Reactions were stopped with 100 µL of 1 M phosphoric acid, and absorbance at 450 nm (OD450) was measured on an Emax microplate reader (Eurofins MWG Operon, Ebersberg, Germany).

### Statistical analysis

Statistical analyses were carried out using GraphPad Prism (version 10.2.0; GraphPad Software, USA). Data were analyzed by one-way ANOVA followed by Bonferroni’s multiple comparison test.

### *In silico* analysis of gB sequences and structures

In order to conduct a sequence conservation analysis, a selection of 475 unique full-length amino acid sequences was downloaded from the NCBI database (search terms: “Human betaherpesvirus 5” [Organism] AND “glycoprotein B“ [Title], with sequence length restricted to 900–910 amino acids, accessed February 2026). Structure analysis and visualization was performed with RasMol (70) and RasTop (https://www.geneinfinity.org/rastop/).
